# Delayed Processing of Chilled Whole Blood for 24 Hours Does Not Affect the Concentration of the Majority of Micronutrient Status Biomarkers

**DOI:** 10.1093/jn/nxab267

**Published:** 2021-09-06

**Authors:** Kerry S Jones, Sarah R Meadows, Karen Chamberlain, Damon A Parkington, Dave Collins, Polly Page, Albert Koulman

**Affiliations:** Nutritional Biomarker Laboratory, Medical Research Council Epidemiology Unit, University of Cambridge, Cambridge, UK; Nutritional Biomarker Laboratory, Medical Research Council Epidemiology Unit, University of Cambridge, Cambridge, UK; Medical Research Council Elsie Widdowson Laboratory, Cambridge, UK; Medical Research Council Elsie Widdowson Laboratory, Cambridge, UK; Nutritional Biomarker Laboratory, Medical Research Council Epidemiology Unit, University of Cambridge, Cambridge, UK; Medical Research Council Epidemiology Unit, University of Cambridge, Cambridge, UK; Medical Research Council Elsie Widdowson Laboratory, Cambridge, UK; Medical Research Council Epidemiology Unit, University of Cambridge, Cambridge, UK; Nutritional Biomarker Laboratory, Medical Research Council Epidemiology Unit, University of Cambridge, Cambridge, UK

**Keywords:** nutrition surveys, nutritional assessment, NDNS, vitamin, micronutrients

## Abstract

**Background:**

The measurement of micronutrient status is essential to understand the health of individuals and populations, but there are limited data on the stability of micronutrients in whole blood.

**Objectives:**

The objective was to investigate the effects of delayed processing of whole blood on the stability of 25 micronutrient and selected clinical biomarkers.

**Methods:**

Blood from 16 healthy adults was collected into EDTA, lithium heparin (LH), or serum tubes. Samples were processed within 2 hours of collection (“2-hour processed”) or mailed overnight (boxed with frozen cold packs) before processing (“24-hour processed”). Micronutrient and clinical biomarker concentrations were quantified with validated methods. The concentration percentage difference between the 2- and 24-hour processed samples was calculated and was compared against quality specifications determined from intra- and interindividual variations.

**Results:**

All analytes had a sample type where the percentage difference concentration between 2-hour and 24-hour processed samples was ≤4% and was acceptable based on calculated limits, including for biomarkers of vitamin A, vitamin D, thiamin, folate, vitamin B-12, iron (ferritin), and zinc status and for selected clinical markers, C-reactive protein, HDL and total cholesterol, and triglycerides. EDTA plasma vitamin C was lower compared to the 2-hour processed sample (geometric mean, 43%; 95% CI: 36%–49%). Pyridoxal-5-phosphate (vitamin B-6 biomarker) decreased, with differences from the 2-hour processed samples of −8% (95% CI: −13% to −2%) and −14% (95% CI: −18% to −9%) in LH plasma and serum, respectively.

**Conclusions:**

In blood collected from adult participants, delayed processing of chilled whole blood for 24 hours did not materially affect the measured concentrations of the majority of micronutrients and selected clinical biomarkers. This suggests that for these analytes, adherence to a 2-hour processing protocol may be unnecessary. This knowledge is valuable and may help to simplify logistics for sample transport and processing of blood samples for micronutrient status assessment.

## Introduction

The measurement of micronutrients is essential to understand the nutritional status of individuals and populations. It enables the assessment of micronutrient concentrations in relation to health and the monitoring and evaluation of micronutrient supplementation and fortification programs. Estimates suggest that micronutrient deficiencies are prevalent in most populations across the world. However, it is also acknowledged that reliable data on micronutrient status are lacking in many countries and populations (1, 2) partly due to the costs and logistical challenges of collecting and processing biological samples.

With the exception of the measurement of hemoglobin for the assessment of anemia, field-based or point-of-care-based methods to assess micronutrient status are not widely used. Point-of-care or lab-on-a-chip technology, including methods that utilize smartphones, are at a relatively early stage of development, including for 25-hydroxyvtiamin D (25OHD) (3), retinol-binding protein and ferritin (4), and vitamin B-12 (5). None of these methods are yet sufficiently validated for application in routine or large studies. The collection and use of capillary blood samples such as dried blood spots (DBS) for micronutrient biomarker analysis negates some of the limitations of venous blood sampling and can simplify sample transport logistics. DBS methods have been published for a limited number of micronutrient biomarkers [reviewed by Verstraete et al. (6)] and have been applied in a number of studies for vitamin A (retinol-binding protein) (7, 8) and vitamin D (9, 10). However, venous blood samples remain the biological sample of choice to ensure reliable, precise, and accurate assessments of micronutrient statuses, using reference methods with established cutoffs for deficiency (2). Consequently, there remains a need to transport biological samples to regional or central laboratories for processing and/or analysis, and this is a particular challenge in rural areas and low- and middle-income countries. Multiple sample types are also typically required to assess a range of micronutrients.

Preanalytical factors associated with sample collection, transport, and processing are major sources of variation in bioanalytical results (11). Guidelines suggest a maximum time of 2 hours before centrifugation of whole blood to prevent significant changes in analyte concentrations (including potassium, phosphorus, glucose, HDL cholesterol, albumin) (12). Consequently, despite limited data, the conservative norm in nutrition-related human studies and surveys is to process whole-blood samples within 2 hours of collection. For example, in the UK National Diet and Nutrition Survey (NDNS), blood samples are taken to hospital or private laboratories located within a 2-hour drive of the participants’ home (13). Whilst this protocol minimizes the time before sample processing, it is logistically challenging and there may be variability between laboratories in sample processing. Quantifying the impact of delayed sample processing for micronutrient status markers is important to clinical practice and population health. Removal of the requirement for rapid blood processing could reduce costs and simplify logistics, enabling sample transport to central laboratories with advantages for standardization of sample processing.

There are data on the effect of preanalytical factors and their impacts on commonly measured clinical analytes, which have included a small number of micronutrient biomarkers (14–16). A few studies have considered the effects of delayed processing on a range of micronutrient biomarkers (17–19). However, in general, whilst micronutrient stability is greater at a cooled rather than room temperature, studies lack sufficient breadth and consistency to allow the development of a generalized approach for micronutrient biomarkers.

Here, we investigate the effect of delayed processing on micronutrient biomarker concentrations. We compare the concentrations of 25 micronutrients and other clinical biomarkers in samples collected, processed, and frozen within 2 hours to samples collected and mailed overnight (chilled and in the dark) before processing and in 3 blood tube types: EDTA and lithium heparin (LH) anti-coagulant and serum.

## Methods

### Study design

The study was conducted from the Medical Research Council Elsie Widdowson Laboratory (MRC EWL; Cambridge, UK). Participants were male and female MRC EWL staff who voluntarily agreed to take part in the study, which was approved by the Cambridge Local Research Ethics Committee. Informed, written consent was obtained. Blood samples were collected by venipuncture over a 2-week period, in the morning (between 08:00 and 10:00), from 16 overnight-fasted participants, into 6 blood tubes, 2 each of EDTA, trace-element free LH, and serum tubes (Sarstedt Monovettes, Sarstedt Ltd). After collection, samples remained at room temperature prior to further processing and aliquots of whole blood were removed for whole-blood folate analysis. The remaining samples were either processed within 2 hours of collection (to mimic processing of samples collected as part of the NDNS Rolling Programme) (13) or packaged and mailed using a next-day, before 09:00 delivery service (www.royalmail.com). Samples for mailing were packed in an Icertech insulated foil box containing 2 Easi-chill cool packs (Icertech) frozen overnight at −20°C. Samples from each participant were mailed separately. The samples mailed overnight were processed after delivery to MRC EWL, approximately 24 hours after the samples were collected from participants. In the manuscript, sample treatments are subsequently referred to as 2-hour or 24-hour processed samples. Aliquots for whole-blood folate analysis were subject to a different protocol that reflected NDNS fieldwork protocols in place at the time (13). Whole-blood folate aliquots were either mailed at the ambient temperature or as per the protocol for the 24-hour processed samples. Further details of methods and the results for this comparison can be found in the **Supplemental Methods** and **Supplemental Results**, respectively.

### Sample processing and analysis

Remaining whole blood samples were centrifuged at 2000 × *g* at 4°C for 20 minutes. Plasma and serum were removed into aliquots in 2 mL microtubes (Sarstedt Ltd.) and stored at −70°C. For vitamin C stabilization, 300 μL of plasma or serum was added to 300 μL of 10% w/v metaphosphoric acid.

After removal of plasma, washed erythrocytes were prepared by adding an approximate volume of normal saline to equal the original whole-blood volume in the tube, gentle inversion, and centrifugation at 2000 × *g* at 4°C for 10 minutes. The supernatant was removed and the process was repeated twice more (20). Washed erythrocytes were stored at −70°C until analysis.

**Table 1** provides a list of analytes and analytical methods. All analyses were performed at the MRC EWL except for whole-blood folate (US CDC) and vitamin B-12 (Addenbrooke's Hospital, Cambridge, UK). Method details are provided in the Supplemental Methods. Contemporaneous quality control and quality assurance data are available (13).

**TABLE 1:** Analytical methods and variation, biological variation, and quality specifications for nutritional and selected clinical biomarkers

		Variation, %^3^			Quality specifications, %^4^		
Analyte	Method^1^	Analytical^2^	Interindividual	Intraindividual	Optimum	Desirable	Minimum
Clinical markers							
C-reactive protein	Clinical Chemistry Analyzer	1.7	76.3^5^	42.2^5^	10.9	21.8	32.7
Ferritin		4.2	15.0^5^	14.9^6^	2.6	5.3	7.9
Creatinine		0.6	18.7^6^	6.8^6^	2.5	5.0	7.5
Triglycerides		0.9	56.8^6^	28.8^6^	8.0	15.9	23.9
HDL cholesterol		1.6	28.3^6^	12.4^6^	3.9	7.7	11.6
Total cholesterol		1.2	22.3^6^	8.2^6^	3.0	5.9	8.9
Fat-soluble vitamins							
25-hydroxyvitamin D	LC-MS/MS	3.3	37.9^6^	11.3^5^	4.9	9.9	14.8
Retinol	HPLC	4.1	30.7^6^	9.5^6^	4.0	8.0	11.6
α-tocopherol		3.6	35.1^6^	11.3^6^	4.6	9.2	13.8
γ-tocopherol		6.7	51.0^6^	12.1^6^	6.6	13.1	19.7
Lutein		5.7	46.0^6^	17.4^6^	6.1	12.3	18.4
β-cryptoxanthin		5.7	58.8^6^	20.5^6^	7.8	15.6	23.4
Lycopene		7.5	47.4^6^	26.1^6^	6.8	13.5	20.3
α-carotene		6.9	54.3^7^	35.8^6^	8.1	16.3	24.4
β -carotene		5.6	67.4^6^	24.2^6^	9.0	17.9	26.9
Minerals							
Selenium	ICP-MS	5.6	13.2^6^	5.1^6^	1.8	3.5	5.3
Zinc		4.2	12.7^7^	9.3^8^	2.0	3.9	5.9
Water-soluble vitamins							
ETKAC (thiamin)	Enzymatic	2.5	4.8^7^	3.7^9^	0.8	1.5	2.3
EGRAC (riboflavin)	Enzymatic	1.5	13.7^7^	3.2^9^	1.8	3.5	5.3
PLP (vitamin B-6)	HPLC	5.0	34.0^6^	20.0^5^	4.9	9.9	14.8
PA (vitamin B-6)		5.0	66.8^7^	67.0^10^	11.8	23.7	35.5
Total folate	LC-MS/MS	2.8	64.3^6^	22.6^6^	8.5	17.0	25.6
Red cell folate	Microbiological	6.5	35.8^6^	9.1^5^	4.6	9.2	13.9
Vitamin B-12	Immunoassay	3.6	43.6^6^	13.4^6^	5.7	11.4	17.1
Holotranscobalamin	ELISA	4.2	41.8^7^	13.0^11^	5.5	10.9	16.4
Vitamin C	Fluorescence	3.4	31.0^6^	26.0^6^	4.4	8.7	13.1

1 Assay equipment or principles are listed in the table. Full details of the methods can be found in the Supplemental Methods. Abbreviations: EGRAC, erythrocyte glutathione reductase activation coefficient ETKAC, erythrocyte transketolase activation coefficient; ICP-MS, inductively coupled plasma mass spectrometry; PA, 4-pyridoxic acid; PLP, pyridoxal-5-phosphate.

2 Analytical precision was obtained from methods as used in the UK National Diet and Nutrition Survey Rolling Programme (13).

3 The inter- and intraindividual percentage variations were used to calculate the quality specifications. Data sources are described in relevant footnotes.

4 Quality specifications were derived from inter- and intraindividual variations, as described by Fraser et al. (22).

5 Chen et al. (21).

6 Lacher et al. (47).

7 UK National Diet and Nutrition Survey (13).

8 González-Revaldería et al. (48).

9 van Dokkum et al. (49).

10 Bor et al. (50).

11 Brokner et al. (51).

### Statistical analysis

To normalize skewed data and to allow calculation of the percentage difference between treatment and sample type, an analysis was performed using log transformed values. The effects of treatment (2-hour or 24-hour processing) and sample type (EDTA/LH/serum) were investigated using a linear mixed model with a random effect of participant ID and fixed effects of treatment type and sample type, with a Sattherwaite adjustment to determine denominator degrees of freedom (used for small sample sizes with unequal variance) (21). Predicted geometric mean concentrations (95% CI) were calculated and pairwise comparisons were made using Stata's post hoc “margins” command (Stata 15; StataCorp LLC). A result was considered significant at a *P* value < 0.05.

The percentage difference between geometric means was calculated by: *1*) exponentiation of the numerical difference between the groups to obtain the ratio difference; and *2*) calculation of the percentage difference: (ratio difference − 1)×100.

The percentage difference of the analyte concentrations of 24-hour from 2-hour processed samples was compared against change limits (optimum, desirable, or minimum performance) calculated from intra- and interindividual variations in a method developed by Fraser et al. (22) and applied by Chen et al. (21). An alternative method to calculate the change limit was also investigated, which used analytical and intraindividual variation (14, 18); however, limits were less stringent than those obtained with the Fraser method. Values for interindividual variation were obtained from various sources, as published by Chen et al. (21) or from the NDNS Rolling Programme (13); the smallest values were chosen in order to produce narrower quality specification limits and to improve confidence in the final outcomes. Analytical precision was obtained from analytical methods as used for the analysis in NDNS (13). Intraindividual variation was obtained from published sources (Table 1).

Raw concentration data were also examined between treatment and sample type by the use of Deming regression plots (Graphpad Version 9; Graphpad Software) and are presented in the Supplemental Results (**Supplemental Figures S1**–**S26**).

## Results

Data were available for up to 16 participants for each analyte, dependent on the number of blood tubes and volume of blood collected. The numbers of data points for each analyte, treatment, and sample type are detailed in Supplemental Results (**Supplemental Table S1**).

### Effect of delayed processing

**Table 2** shows the percentage difference between treatments for each analyte and sample type. For vitamin B-6, the geometric mean concentrations of pyridoxal-5-phosphate (PLP) in LH plasma and serum of 24-hour processed samples were significantly lower than those in the 2-hour processed samples [−8% (95% CI: −13 to −2) and −14% (95% CI: −18 to −9), respectively]. The −14% difference for serum was at the minimum quality specification (14.8%; Table 1).

**TABLE 2:** Geometric mean percentage difference in analyte concentration and relation to quality specifications between whole-blood samples processed within 2 hours or after 24 hours^1^

	Geometric mean percent difference (95% CI) from 2-hour processing		
	Sample type		
Analyte	EDTA	LH	Serum
Clinical markers			
C-reactive protein	3 (−11 to 20)	0.5 (−13 to 16)	−3 (−16 to 11)
Ferritin	2 (−0.3 to 4)	1 (−1 to 3)	0.3 (−2 to 2)
Creatinine	1 (−2 to 4)	0 (−3 to 3)	0.7 (−2 to 3)
Triglycerides	−2 (−8 to 5)	2 (−4 to 9)	0.1 (−6 to 6)
HDL cholesterol	−0.1 (−2 to 2)	−2 (−4 to −1)^2^	−1 (−2 to 1)
Total cholesterol	−0.1 (−2 to 2)	−1 (−2 to 1)	0.4 (−1 to 2)
Fat-soluble vitamins			
25-hydroxyvitamin D	2 (3–7)	−1 (−6 to 3)	−4 (−8 to 0.2)
Retinol	−0.4 (−7 to 7)	−0.2 (−7 to 7)	2 (−4 to 9)
α-tocopherol	1 (−4, 6)	−1 (−6, 4)	1 (−4, 5)
γ-tocopherol	2 (−3 to 7)	−0.6 (−5 to 4)	2 (−3 to 7)
Lutein	1 (−5 to 8)	−1 (−7 to 5)	2 (−4 to 9)
β-cryptoxanthin	1 (−4 to 7)	−4 (−9 to 1)	−1 (−6 to 5)
Lycopene	3 (−3 to 9)	−3 (−8 to 2)	−2 (−7 to 4)
α-carotene	2 (−4 to 9)	−6 (−11 to 1)	−5 (−11 to 1)
β -carotene	4 (2–9)	−3 (−8 to 2)	−2 (−7 to 3)
Minerals			
Selenium	−1 (−3 to 2)	−1 (−3 to 1)	−1 (−3 to 1)
Zinc	1 (−2 to 3)	6 (3–8)^2^, ^5^	1 (−1 to 4)
Water-soluble vitamins			
ETKAC (thiamin)	−1 (−3 to 1)^3^	−0.3 (−2 to 2)	n/m
EGRAC (riboflavin)	0 (−2 to 2)	−1 (−3 to 0.6)	n/m
PLP (vitamin B-6)	4 (−2 to 10)	−8 (−13 to −2)^2^, ^3^	−14 (−18 to −9)^2^, ^4^
PA (vitamin B-6)	1 (−5 to 7)	3 (−2 to 9)	−5 (−10 to 0.5)
Total folate	−2 (−5 to 1)	−2 (−5 to 1)	1 (−2 to 4)
Vitamin B-12	−3 (−6 to −0.4)^2^	0.2 (−3 to 3)	−2 (−4 to 1)
Holotranscobalamin	9 (2–16)^2^, ^3^	5 (−1 to 12)	−4 (−9 to 2)
Vitamin C	−43 (−49 to −36)^2^, ^5^	2 (−9 to 14)	0.1 (−11 to 12)

1 The 24-hour processed samples were mailed, cooled, overnight after collection, and processed on arrival at the laboratory. All analytes were measured in plasma or serum except for ETKAC and EGRAC (measured in saline-washed red blood cells). Percent differences were calculated from a linear mixed model with the random effect of participant ID and fixed effects of treatment and sample type. Quality specifications for bias were calculated from intra- and interindividual variations (22). No superscript indicates better than optimum performance. Abbreviations: EGRAC, erythrocyte glutathione reductase activation coefficient; ETKAC; erythrocyte transketolase activation coefficient; LH, lithium heparin plasma; n/m; not measured, sample type not suitable for analysis; PA, 4-pyridoxic acid; PLP, pyridoxal-5-phosphate.

2 Significant concentration difference (*P* < 0.05) from samples that underwent processing within 2 hours of collection.

3 Percent difference between treatments is between optimal and desirable performance specifications.

4 Percent difference between treatments is between desirable and minimum performance specifications.

5 Percent difference between treatments is worse than minimum performance specification.

For the clinical markers in 24-hour processed samples, percentage differences from the 2-hour processed samples were ≤3% in all sample types and were within the optimum quality specifications, although there was a significant −2% (95%CI: −4 to −1) difference observed for HDL cholesterol in LH plasma.

Enzymatic activity coefficients of thiamin (ETKAC) and riboflavin (EGRAC) were up to 1% lower in 24-hour processed samples; for ETKAC, this small difference fell between the optimum and desirable quality specifications but was not consistent between sample types.

Differences for vitamin B-12 were within the optimum quality specifications, although the vitamin B-12 concentrations were significantly lower (−3%; 95% CI: −6 to −0.4) in the EDTA 24-hour processed samples. Holotranscobalamin and vitamin C concentrations in LH plasma and serum were not different between treatments. However, in EDTA plasma, the holotranscobalamin concentration was significantly higher (9%; 95% CI: 2%–16%) and the vitamin C concentration was significantly lower (−43%; 95% CI: −49% to −36%) in 24-hour processed samples compared to 2-hour processed samples.

Selenium and zinc concentrations were not different between treatments, except for zinc in LH plasma, which was significantly higher in the 24-hour processed sample (5.7%; 95% CI: 3%–8%), a value at the minimum quality specification (5.9%; Table 1).

There were no significant differences between sample treatments for 25OHD, retinol, tocopherols, carotenoids, or total folate, and numerical differences were within the optimum quality specifications.

### Effect of sample type

**Table 3** details the geometric mean concentration (95% CI) for each analyte by treatment and sample type, highlighting significant (*P* < 0.05) differences. Total and HDL cholesterol were significantly lower in EDTA plasma compared to LH plasma or serum, whilst C-reactive protein was significantly lower in LH plasma, equal to a 38% (95% CI: 19%–59%) difference from serum in 2-hour processed samples.

**TABLE 3:** Geometric mean concentration (95% CI) by treatment (processed within 2 hours or 24 hours) and sample type (EDTA, LH, or serum)^1^, ^2^

	2-hour processing			24-hour processing		
	Sample type			Sample type		
Analyte	EDTA	LH	Serum	EDTA	LH	Serum
Clinical markers						
C-reactive protein, mg/L	1.75 (1.14–2.70)^a^	1.45 (0.94–2.24)^b^	2.00 (1.30–3.07)^a^	1.81 (1.17–2.78)^a^	1.46 (0.95–2.24)^b^	1.93 (1.26–2.97)^a^
Ferritin, μg/L	47 (30–72)^a^	49 (32–76)^b^	49 (32–76)^b^	48 (31–74)^a^	49 (32–76)^b^	49 (32–76)^b^
Creatinine, μmol/L	72.8 (68.0–78.0)^a^	75.8 (70.8–81.1)^b^	75.7 (70.8–81.0)^b^	73.8 (69.0–79.0)^a^	75.8 (70.8–81.1)^b^	76.2 (71.2–81.5)^b^
Triglycerides, mmol/L	1.21 (0.88–1.65)	1.16 (0.85–1.58)	1.18 (0.86–1.61)	1.19 (0.87–1.62)	1.18 (0.87–1.62)	1.18 (0.86–1.61)
HDL cholesterol, mmol/L	1.30 (1.31–1.59)^a^	1.38 (1.13–1.68)^b^	1.38 (1.13–1.68)^b^	1.30 (1.06–1.58) ^a^	1.35 (1.10–1.64)^2^^,b^	1.36 (1.12–1.67) ^b^
Total cholesterol, mmol/L	4.72 (4.28–5.20)^a^	4.86 (4.41–5.35)^b^	4.92 (4.46–5.42)^b^	4.71 (4.28–5.19)^a^	4.83 (4.38–5.32)^b^	4.94 (4.48–5.44)^c^
Fat-soluble vitamins						
25-hydroxyvitamin D, nmol/L	32 (25–41)^a^	34 (26–43)^a,b^	35 (27–44)^b^	33 (25–42)	33 (26–42)	33 (26–43)
Retinol, μmol/L	1.43 (1.30–1.58)^a^	1.57 (1.42–1.73)^b^	1.53 (1.38–1.69)^a,b^	1.43 (1.29–1.58)^a^	1.57 (1.42–1.73)^b^	1.57 (1.42–1.73)^b^
α-tocopherol, μmol/L	24.1 (21.5–27.1)^a^	26.0 (23.2–29.2)^b^	25.6 (22.8–28.8)^b^	24.4 (21.7–27.4)^a^	25.7 (22.9–28.9)^b^	25.8 (23.0–29.0)^b^
γ-tocopherol, μmol/L	1.30 (1.16–1.47)^a^	1.40 (1.24–1.57)^b^	1.38 (1.23–1.55)^b^	1.33 (1.18–1.49)^a^	1.39 (1.24–1.57)^a,b^	1.41 (1.25–1.58)^b^
Lutein, μmol/L	0.33 (0.27–0.40)^a^	0.37 (0.30–0.44)^b^	0.36 (0.29–0.43)^b^	0.33 (0.28–0.40)^a^	0.36 (0.30–0.44)^b^	0.36 (0.30–0.44)^b^
β-cryptoxanthin, μmol/L	0.19 (0.14–0.26)^a^	0.21 (0.16–0.28)^b^	0.20 (0.15–0.27)^b^	0.19 (0.4–0.26)	0.20 (0.15–0.27)	0.20 (0.15–0.27)
Lycopene, μmol/L	0.87 (0.72–1.05)^a^	0.95 (0.78–1.14)^b^	0.92 (0.76–1.11)^a,b^	0.89 (0.74–1.08)	0.92 (0.76–1.10)	0.91 (0.75–1.09)
α-carotene, μmol/L	0.15 (0.12–0.19)^a^	0.17 (0.13–0.21)^b^	0.17 (0.13–0.21)^b^	0.15 (0.12–0.19)	0.16 (0.13–0.20)	0.16 (0.13–0.20)
β -carotene, μmol/L	0.59 (0.40–0.86)^a^	0.64 (0.44–0.94)^b^	0.62 (0.43–0.91)^b^	0.61 (0.42–0.89)	0.62 (0.43–0.91)	0.61 (0.42–0.89)
Minerals						
Selenium, μg/L	77 (72–82)^a^	80 (75–85)^b^	79 (74–84)^b^	76 (72–81)^a^	80 (75–85)^b^	79 (74–84)^b^
Zinc, μg/L	812 (778–847)	800 (767–834)	802 (769–837)	817 (783–853)^a^	844 (810–881)^2^^,b^	812 (779–847)^a^
Water-soluble vitamins						
ETKAC (thiamin) [ratio]	1.11 (1.08–1.15)	1.10 (1.07–1.13)	n/m	1.10 (1.07–1.14)	1.10 (1.07–1.13)	n/m
EGRAC (riboflavin) [ratio]	1.35 (1.26–1.45)	1.35 (1.26–1.45)	n/m	1.35 (1.26–1.45)	1.34 (1.25–1.43)	n/m
PLP (vitamin B-6), nmol/L	51.9 (39.7–67.7)	49.6 (38.0–64.8)	52.1 (39.9–68.0)	54.0 (41.4–70.5)^a^	45.9 (32.5–59.9)^2^^,b^	44.9 (34.4–58.6)^2^^,b^
PA (vitamin B-6), nmol/L	20.1 (15.9–25.3)^a^	20.9 (16.6–26.3)^a,b^	21.5 (17.1–27.2)^b^	20.2 (16.1–25.5)	21.4 (17.0–27.0)	20.5 (16.3–25.9)
Total folate, nmol/L	16.2 (12.3–21.4)^a^	17.0 (12.9–22.5)^b^	17.0 (12.9–22.4)^b^	15.9 (12.1–20.9)^a^	16.7 (12.6–21.9)^b^	17.2 (13.0–22.6)^c^
Vitamin B-12, ng/L	401 (336–479)^a^	393 (330–469)^a^	380 (319–453)^b^	388 (326–463)^2^^,a^	394 (330–470)^a^	373 (313–445)^b^
Holotranscobalamin, pmol/L	68 (55–84)^a^	63 (51–78)^b^	77 (62–95)^c^	74 (60–91)^2^^,a^	66 (53–82)^b^	74 (60–91)^a^
Vitamin C, μmol/L	55 (45–67)	58 (48–71)	57 (47–70)	31 (26–38)^2^^,a^	59 (49–72)^b^	58 (47–70)^b^

1 The 24-hour processed samples were mailed, cooled overnight after collection, and processed on arrival at the laboratory. All analytes were measured in plasma or serum except for ETKAC and EGRAC (measured in saline-washed red blood cells). Geometric means with uncommon letters are significantly different (*P* < 0.05) from other sample types within treatment (2-hour or 24-hour processing). Abbreviations: EGRAC, erythrocyte glutathione reductase activation coefficient; ETKAC; erythrocyte transketolase activation coefficient; LH, lithium heparin plasma; n/m, not measured, sample type not suitable for analysis; PA, 4-pyridoxic acid; PLP, pyridoxal-5-phosphate

2 Significantly different from 2-hour processed sample.

Concentrations of retinol, carotenoids, selenium, and total folate were consistently lower in EDTA plasma. Retinol concentrations were 7% (95% CI: 0%–14%) lower in EDTA plasma compared to serum in 2-hour processed samples and 10% (95% CI: 2%–17%) lower in both serum and LH plasma in 24-hour processed samples. Similarly, in 2- and 24-hour processed samples, α-tocopherol was between 6% and 8% lower (*P* < 0.05) in EDTA plasma than LH plasma or serum, but there was no difference between serum and LH plasma.

Total folate was significantly lower in EDTA plasma in both 2-hour and 24-hour processed samples compared with LH plasma [difference 5% (95% CI: 2%–8%) for each] and serum [differences: 5% (95% CI: 2%–8%) and 8% (95% CI: 5%–11%) for 2- and 24-hour processed samples, respectively]. This is primarily due to lower 5-methyltetrahydrofolate (5-MTHF; **Supplemental Table S2**) and, in the 24-hour processed samples, higher MeFox (pyrazino-s-triazine derivative of 4α-hydroxy-5MTHF, an oxidation product of 5-MTHF; **Supplemental Table S3**). Vitamin B-12 was significantly lower in serum than either EDTA (−5%; 95% CI: −8% to −3%) or LH plasma (−3%; 95% CI: −6% to −1%; in 2-hour samples). For 2-hour processed samples, significant differences were apparent between all sample types for holotranscobalamin with concentrations in the ranking of serum > EDTA > LH, with the largest difference (23%; 95% CI: 15%–31%) between serum and LH plasma. There were no differences between sample types for zinc, EGRAC, ETKAC, vitamin B-6, or vitamin C for the 2-hour processed sample.

In contrast to results for the 2-hour processed samples, the PLP concentration in the 24-hour processed samples was significantly higher in EDTA plasma [15% (95% CI: 10%–19%) compared with LH; 17% (95% CI: 12%–21%) compared with serum] and vitamin C was significantly lower in EDTA plasma [-88% (95% CI: −112% to −67%) compared with LH; −83% (95% CI: −106% to −63%) compared with serum]. Similarly, the zinc concentration was marginally but significantly higher in LH samples in the 24-hour processed results compared to both EDTA plasma (3%; 95% CI: 0.5%–6%) and serum (4%; 95% CI: 1%–6%).

## Discussion

This study investigated the effects of delayed processing of whole blood, kept chilled and in 3 blood tube types, on a range of nutritional biomarkers and selected clinical markers typically measured in nutritional surveys and studies. For the majority of analytes, including those recognized as being of the greatest global public health concern (retinol, thiamin, folate, vitamin B-12, vitamin D, ferritin, and zinc), there was no significant effect of delayed processing for at least one sample type. Differences in concentration were within a few percentages of samples processed shortly after collection and were within optimal performance specifications calculated from normal biological variation. This suggests that for these analytes, studies and surveys do not need to adhere to a 2-hour processing protocol. This knowledge may be beneficial in terms of simplifying logistics for sample transport and processing at a central laboratory.

Existing data on nutritional biomarker stability in unprocessed whole blood are relatively sparse. The ranges of temperatures and time conditions employed in different studies; differences in analytical, data, and statistical methods (e.g., acceptance criteria); and data presentation can make definitive conclusions difficult to draw. Studies primarily focusing on clinical analytes (14–16, 23–25) have included a small number of nutritional biomarkers (e.g., folate and vitamins A and B-12) and reported stability within the acceptance criteria set for these studies. However, acceptance criteria can be relatively wide; for example, Zhang et al. (15) observed changes of ∼15% for serum folate, within their clinical acceptance limit but outside their specified analytical limit. Whilst wider limits may be workable in a clinical setting, they may not be applicable to population nutritional surveillance for monitoring micronutrient status over time or between different surveys.

Two studies have focused on a broad range of nutritional biomarkers. Cuerq et al. (18) examined the stability of vitamins A, E, K, B-6, B-12, and C; thiamin; riboflavin; folate; and a selection of carotenoids in whole blood for up to 48 hours at room temperature and observed significant decreases in folate and vitamin C and an increase in vitamin B-6. Drammeh et al. (17) studied change in analytes in serum from whole blood maintained at 32°C for 1, 2, or 3 days and observed generally good stability, with the exceptions of ferritin and folate from 1 day onwards and for retinol after 3 days.

One of the largest decreases we observed was for PLP, a biomarker of vitamin B-6 status, with 8% and 14% decreases in the PLP concentrations measured in LH plasma and serum, respectively. This decrease was not apparent in EDTA plasma. PLP is known to be relatively sensitive to light and temperature (26), and studies have shown decreases in PLP concentrations after delayed processing of whole blood (27) and in plasma or serum at room temperature (28, 29). In contrast, other studies have reported little or no difference (30, 31) or even increases in PLP concentrations with delayed processing (18) in serum and plasma (32) at room temperature and with refrigeration (29). These differing conclusions may be due in part to the combinations of test conditions, anticoagulants, and methods used. Rybak and Pfeiffer (26) have described an interfering peak in the HPLC chromatograms of EDTA plasma that may be quantified as PLP and may explain the apparent stability in our study (which used the Rybak method), as well as in other studies (18).

Our data show that plasma/serum total folate, measured by LC-MS/MS, was stable under the conditions tested; the small percentage decrease in the 24-hour samples compared to the 2-hour samples was not significant and was within the target specifications. Other studies have shown that delayed processing of whole blood kept at room temperature or 32°C for 1–2 days led to decreases in serum folate of between 9% and 17% (17, 18). Our data indicate that cooling can mitigate the losses associated with delayed processing of whole-blood samples for serum/plasma total folate analysis and are consistent with the recommendations published by the Biomarkers of Nutrition for Development consortium (33). We observed a significantly lower total folate concentration in EDTA plasma compared with LH plasma and serum. Similar differences in folate concentrations between EDTA plasma and serum have been observed (34) and may be associated with the faster degradation of folate associated with EDTA samples (35). Consistent with this observation, we observed lower 5-MTHF and higher MeFox concentrations in the EDTA plasma of 24-hour processed samples compared to 2-hour processed samples and in EDTA plasma compared to other sample types (Supplementary Tables S2–S3). Consequently, our study endorses the recommendation (35) that EDTA plasma is not recommended for the assessment of plasma folate.

We show that the fat-soluble vitamins (specifically, vitamins A, D, and E and the carotenoids) in whole blood are stable after delayed processing when chilled and that concentrations in plasma and serum are comparable to those obtained from samples processed within 2 hours. These data are consistent with the studies described above (14, 17, 18) and with studies that focused specifically on fat-soluble vitamins in whole blood stored at +4°C and +20°C and for 24+ hours (19, 36–38), as well as relevant laboratory best-practice guidelines (39). For retinol, whilst Key et al. (36) observed a significant 3% decrease in serum retinol after 24 hours at 4°C, our study and other studies (17–19, 37) observed no statistically significant change. We also observed significantly lower retinol concentrations in ETDA plasma compared to LH plasma in 2-hour processed samples and compared to both LH plasma and serum in 24-hour processed samples. These results are consistent with data that have shown no difference between serum and LH plasma (40) but lower concentrations in EDTA plasma (41), and suggest that EDTA plasma may not be a suitable sample type for assessing plasma retinol.

There was no difference in vitamin C concentrations between blood tube types when samples were processed within 2 hours, suggesting vitamin C can be measured in EDTA or LH plasma or serum if samples are processed soon after collection. Vitamin C instability is well known, and rapid processing, refrigeration, and stabilization with the addition of acid is recommended (18). However, we observed that whilst vitamin C concentrations in serum and LH plasma were not affected by delayed processing, the concentration in EDTA plasma from 24-hour processed samples decreased by almost half. Pullar et al. (42) also observed decreases in vitamin C concentrations in EDTA whole blood over 24 hours, whether stored on ice or at room temperature. Other studies have also reported greater losses in EDTA whole blood than serum or LH plasma (36, 43). These data suggest that EDTA as an anticoagulant is best avoided for the analysis of vitamin C if blood samples are not processed soon after collection.

In contrast to some studies (44) but in keeping with the conclusions of a recent review (45), we found no difference in zinc concentrations between blood tube type when samples were processed promptly. However, these conflicting results may be affected by the brand and manufacturing processes of different tubes, in addition to the direct effect of anticoagulants. In the 24-hour processed samples, the zinc concentrations appeared stable in EDTA and serum tubes but not LH tubes. Other studies have observed increases in serum zinc concentrations with delayed processing of whole blood stored at 25°C to 30°C (27, 46), probably due to the zinc leakage from cellular components. Our data suggest that in EDTA and serum tubes, but not LH tubes, the increase in zinc concentration with time can be ameliorated with cooling. The reasons for this are unknown. Zinc measurements are particularly sensitive to external contamination. In this study, we used trace element–free LH tubes, but serum and EDTA tubes were not certified as being free of trace elements.

Our results show that the stability of many biomarkers of nutritional status is not a concern, under the conditions tested, for the resulting data to be used with confidence in clinical situations, studies, and population surveys. In general, and as discussed for individual analytes above, there were fewer differences in biomarker concentrations between LH and serum tubes compared to results from samples collected in ETDA tubes. The choice of sample type will be influenced not only by the biomarker sample stability but also determined by the logistics, costs, and practicalities of fieldwork and sample collection. Compromises in the choice of sample type may be required, particularly where there is a large panel of measures.

The analytical methods used in this study were well established, with rigorous quality control (13) and, where available, external quality assurance. However, there are a number of limitations. Although our findings have general applications, they were obtained under a specific set of experimental conditions, including population, study design, and analytical methods. Additional studies may be necessary to quantify the effects of delayed processing in other populations, conditions (duration and temperature), and fieldwork and laboratory infrastructures. In addition, observations around sample stability in different blood collection tubes may be specific to the study and method, and further evaluation for other assays may be required.

Quality specification calculations were performed with values for intraindividual variation obtained from a number of different sources with assays that may not have been the same as those used in this analysis; it is unknown whether there are method-specific differences in intraindividual variation. We measured healthy adults only and results may be different for other population groups (e.g., children, pregnant women, or patients) or may be concentration dependent: for example, Henriksen et al. (16) observed a concentration-dependent effect on the stability of HDL cholesterol. Although the conditions were standardized for all samples tested, we did not record the temperatures of samples on delivery or the precise duration of the delay in sample processing of the mailed samples.

The control of preanalytical variation is a critical component for the provision of reliable nutritional biomarker data. In this study, we have shown that for a broad range of nutritional biomarkers and selected clinical markers, delayed processing of chilled whole blood for 24 hours does not materially affect the measured concentrations in at least one of the sample types tested. PLP was the least stable analyte, and additional precautions may be required for the reliable assessment of vitamin B-6 status.

## Supplementary Material

nxab267_Supplemental_Files

## Notes

At the Medical Research Council Elsie Widdowson Laboratory the study was funded by the UK Medical Research Council, programme U105960384. KSJ, SRM, DAP, PP, DC, and AK are supported by the National Institute for Health Research (NIHR) Cambridge Biomedical Research Centre (BRC) (IS-BRC-1215- 20014), comprising the NIHR BRCNutritional Biomarker Laboratory and NIHR BRC Dietary Assessment and Physical Activity Group. The NIHR Cambridge Biomedical Research Centre is a partnership between Cambridge University Hospitals NHS Foundation Trust and the University of Cambridge, funded by the NIHR.

Author disclosures: KSJ, SRM, DAP, PP, DC, and AK are supported by the National Institute for Health Research (NIHR) Cambridge Biomedical Research Centre (IS-BRC-1215- 20014), comprising the NIHR BRCNutritional Biomarker Laboratory and NIHR BRC Dietary Assessment and Physical Activity Group. KC reports no conflicts of interest.

The views expressed are those of the authors and not necessarily those of the NHS, the NIHR or the Department of Health and Social Care.

Supplemental Methods, Supplemental Results, Supplemental Figures 1–26, and Supplemental Tables 1–3 are available from the “Supplementary data” link in the online posting of the article and from the same link in the online table of contents at https://academic.oup.com/jn.

Abbreviations used: DBS, dried blood spots; EGRAC, erythrocyte glutathione reductase activity coefficient; ETKAC, erythrocyte transketolase activity coefficient; LH, lithium heparin; MeFox, pyrazino-s-triazine derivative of 4α-hydroxy-5-methyltetrahydrofolate; MRC EWL, Medical Research Council, Elsie Widdowson Laboratory; NDNS, National Diet and Nutrition Survey; PLP, pyridoxal-5-phosphate; 25OHD, 25-hydroxyvitamin D; 5-MTHF, 5-methyltetrahydrofolate.
