# Supplementary material for: Delayed Processing of Chilled Whole Blood for 24 Hours Does Not Affect the Concentration of the Majority of Micronutrient Status Biomarkers
Source: J Nutr. 2021 Sep 6;151(11):3524–32. doi: 10.1093/jn/nxab267 (PMC8564691; doi:10.1093/jn/nxab267)
Supplement: nxab267_Supplemental_Files [file nxab267_Supplemental_Files.zip › JN-2021-0500_R1 Supplemental data methods.docx]

**Supplemental Methods**

**Summary of analytical methods**

*All methods were performed at MRC Elsie Widdowson Laboratory, Cambridge, UK unless otherwise stated.*

1. **Clinical markers**
   1. **C-reactive protein**

C-reactive protein (CRP) was measured using an extended range assay (RCRP, DF34) on Siemens Dimension Xpand analyser (Siemens Healthcare Limited, Camberley, Surrey, UK). The CRP method is based on a particle enhanced turbidimetric immunoassay (PETIA) technique with a detection range from 0.5 mg/L to 250 mg/L. Latex particles coated with anti-CRP antibody aggregate in the presence of CRP in the sample. The increase in turbidity that accompanies aggregation is proportional to the CRP concentration.

- 1. **Ferritin**

Ferritin was measured on a Siemens Dimension Xpand analyser (Siemens Healthcare Limited, Camberley, Surrey, UK). In the assay, the sample is incubated with chromium dioxide particles coated with specific antibodies to human ferritin and conjugate reagent. The assay is coupled to a colour reaction and the concentration of ferritin in the sample is proportional to the intensity of colour produced in the reaction.

- 1. **Creatinine**

Creatinine was measured with an enzymatic method on a Siemens Dimension Xpand analyser (Siemens Healthcare Limited, Camberley, Surrey, UK) which uses creatininase coupled to creatinase, sarcosine oxidase and peroxidase. The coloured end product is measured using a bichromatic (510 nm, 700 nm) endpoint technique.

- 1. **Triglycerides**

The triglycerides (triacylglycerols) were measured on a Siemens Dimension Xpand analyser (Siemens Healthcare Limited, Camberley, Surrey, UK). In the method, the sample is incubated with lipoprotein lipase enzyme reagent that converts triglycerides into free glycerol and fatty acids. Glycerol kinase catalyses the phosphorylation of glycerol by adenosine-5-triphosphate (ATP) to glycerol-3-phosphate. Glycerol-3-phosphate-oxidase oxidises glycerol-3-phosphate to dihydroxyacetone phosphate and hydrogen peroxide (H_2_O_2_). The catalytic action of peroxidase forms quinoneimine from H_2_O_2_, aminoantipyrine and 4-chlorophenol. The change in absorbance due to the formation of quinoneimine is directly proportional to the total amount of glycerol and its precursors in the sample and is measured using a bichromatic (510/700 nm) endpoint technique.

- 1. **Total cholesterol**

The total cholesterol was measured on a Siemens Dimension Xpand analyser (Siemens Healthcare Limited, Camberley, Surrey, UK). The total cholesterol method (Siemens CHOL DF27) uses cholesterol esterase to catalyse the hydrolysis of cholesterol esters to produce free cholesterol which, along with pre-existing free cholesterol, is oxidised in a reaction catalysed by cholesterol oxidase to form cholest-4-ene-3-one and hydrogen peroxide. In the presence of horseradish peroxidase, the hydrogen peroxide thus formed is used to oxidize N,N-diethylaniline-HCl/4-aminoantipyrine to produce a chromophore that absorbs at 540 nm.

- 1. **HDL cholesterol**

Performed on a Siemens Dimension Xpand analyser (Siemens Healthcare Limited, Camberley, Surrey, UK) the AHDL cholesterol assay (Siemens DF48B) is a method for directly measuring high density lipoprotein (HDL) cholesterol concentrations. The method is based on accelerating the reaction of cholesterol oxidase with non-HDL unesterified cholesterol and dissolving HDL selectively using a specific detergent. In the first reaction, non-HDL unesterified cholesterol is subject to a cholesterol oxidase reaction and the peroxide generated is consumed by a peroxidase reaction with DSBmT yielding a colourless product. The second reagent consists of a detergent capable of solubilising HDL specifically, cholesterol esterase and chromagenic coupler to develop colour that is measured using a bichromatic (600/700 nm) endpoint technique. The colour intensity of the dye is directly proportional to the serum HDL cholesterol concentration.

1. **Fat-soluble vitamins**
   1. **25-hydroxyvitamin D (25OHD)**

The assay for 25-hydroxyvitamin D (25OHD) used a methanol protein precipitation step followed by liquid-liquid extraction in hexane. Stable isotope (deuterium) labelled internal standards were added during the extraction step; d_6_-25-hydroxyvitamin D_3_, d_3_-25-hydroxyvitamin D_2_ and d_3_-epi 25-hydroxyvitamin D_3_ (Sigma Aldrich, St Louis, Missouri, US). Samples were measured with UPLC-MS/MS using a Waters ACQUITY UPLC system (Waters UK, Herts, UK) coupled to an AB Sciex QTrap mass spectrometer (AB Sciex UK Ltd, Cheshire, UK). Analytes were resolved using reversed phase UPLC on a Thermo Scientific Hypersil GOLD PFP 2.1 x 100 mm 1.9 µm column (Thermofisher Ltd, Loughborough, UK) at 40°C with a 72% methanol +0.1% formic acid isocratic mobile phase at 0.25 mL/min prior to mass spectrometry analysis. Total 25OHD was calculated from the sum of 25-hydroxyvitamin D_2_ (25OHD_2_) and 25-hydroxyvitamin D_3_ (25OHD_3_). If a value was between the limit of detection (LoD) and limit of quantification (LoQ) an imputed value of LoQ/√2 was used in the calculation of total 25OHD. If 25OHD_2_ was below the LoD, a value of zero was used and the total 25OHD value was equal to the 25OHD_3_ concentration. The epi-25OHD_3_ was chromatographically separated using this method and so was not included in the total 25OHD. The 25OHD method was calibrated using in-house solvent standards run with every assay and accuracy checks performed using standard reference materials (NIST, Gaithersburg, Maryland, US) and via the vitamin D external quality assessment scheme (DEQAS) scheme (London, UK).

- 1. **Retinol, tocopherol and carotenoids**

The assay for retinol, α- and γ-tocopherol, and individual carotenoids was based on that published by Sowell et al. (1) and used a methanol protein precipitation step followed by liquid-liquid extraction in hexane. Internal standards (tocopherol acetate and apo-8’-carotenal) were added during the extraction step. Detection of retinol, α-tocopherol, γ-tocopherol, lutein and zeaxanthin (combined due to co-elution), lycopene, α-cryptoxanthin, β-cryptoxanthin, α-carotene, β-carotene and internal standards was accomplished by HPLC analysis with PDA (UV, photo diode array) detection (Waters UK, Herts, UK). Analytes were resolved using reversed phase HPLC on a YMC-pack pro C18 4.6 x 150 mm 3 µm column (Crawford Scientific, Lanarkshire, UK) at 40°C with a 25:75 ethanol + 0.1% triethylamine: acetonitrile + 0.1% triethylamine isocratic mobile phase at 1.2 mL/min. The assay was calibrated using in-house solvent standards run with every assay and accuracy checks performed using standard reference materials (NIST, Gaithersburg, Maryland, US). External quality assessment was performed by participantion in the scheme run by UKNEQAS.

1. **Zinc and selenium**

Selenium (Se) and zinc (Zn) concentrations were determined by measuring the ^78^Se and ^68^Zn isotopes using an inductively coupled plasma mass spectrometer (ICP-MS) BRAND equipped with a dynamic reaction cell (DRC). Methane (CH_4_) was used as a DRC gas to overcome argon based interferences. Samples were introduced to the ICP-MS through a flow injection system combined with the Sea spray nebulizer and cyclonic spray chamber arrangement. Samples and QC materials were prepared in diluent which included rhodium as internal standard. Assay performance was assessed by participantion in the Inter-laboratory Comparison Program for Metals in Biological Matrices (PCI), operated by Centre de Toxicologie du Québec at the Institut National de Santé Publique du Québec (INSPQ). ClinChek Plasma Control Lyophilised for Trace Elements Level 1 and 2 (Recipe Chemicals and Instruments GmbH) were used to monitor performance.

1. **Water-soluble vitamins**
   1. **Erythrocyte transketolase activity coefficient (ETKAC) for thiamin (vitamin B1)**

This assay depends on the coupling of pyridine nucleotide oxidation to glycerol phosphate dehydrogenase (GDH) (NADH linked), which produces glycerol-3-phosphate after the transketolase-catalysed conversion of ribose-5-phosphate. The rate of oxidation of NADH is monitored at 340 nm, on the Multiskan FC plate-reader (Thermofisher Ltd, Loughborough, UK). Thiamine status is assessed using the activation coefficient, which is the ratio of cofactor-stimulated activity to the basal activity without any added cofactor with higher values indicated greater deficiency. Further details on the assay are available (2). There are no commercially available calibration materials or EQA schemes available for the assessment of ETKAC. Assay performance was monitored with the use of washed erythrocytes prepared in-house.

- 1. **Erythrocyte glutathione reductase activation coefficient (EGRAC) for riboflavin (vitamin B2)**

The assay was developed from the original manual technique developed by Glatzle et al. (3) and later adapted modified to an assay carried out on microplates and read on a Thermo iEMS plate reader (Thermofisher Ltd, Loughborough, UK). The ratio of flavin adenine dinucleotide (FAD) stimulated to unstimulated erythrocyte glutathione reductase activity is the EGRAC and is a measure of riboflavin status. The method is a kinetic test with decreasing absorbance and the pre-incubation with FAD is carried out for a relatively long period, 30 minutes at 37°C, to ensure full reactivation of apo-enzyme. The assay is conducted at a low final concentration of FAD (1.5 μM), which is necessary to eliminate activation coefficients (ratios) <1.0; this can result from enzyme inhibition by FAD, or its breakdown products, which may occur if the final concentration of FAD is too high. The generally accepted threshold for riboflavin adequacy is EGRAC below 1.30. There are no commercially available calibration materials or EQA schemes available for the assessment of EGRAC. Assay performance was monitored with the use of washed erythrocytes prepared in-house.

- 1. **Pyridoxal-5-phosphate (PLP) and 4-pyridoxic acid (PA) for vitamin B-6 status**

The assay for PLP and PA is based on that published by Rybak & Pfeiffer (4) and uses a metaphosphoric acid protein precipitation followed by filtration of the supernatant. Detection of PLP and PA was accomplished by HPLC with fluorescence detection employing a post-column derivatisation step with sodium chlorite. Analytes were resolved using reversed phase HPLC on a HypersilTM BDS , C18 3 x 150 mm 5 μm column (Thermo Scientific, Loughborough,UK), at 35°C with a gradient mobile phase combining 50 mM sodium dihydrogen phosphate buffer at pH 2.7 plus 0.2% acetonitrile and methanol at 0.7 mL/min. The assay was calibrated using in-house aqueous standards prepared alongside QCs and samples with every batch and accuracy checks performed using standard reference materials (NIST, Gaithersburg, Maryland, US).

- 1. **Serum/plasma total folate**

Serum/plasma folate was measured by liquid chromatography-tandem mass spectrometry (LC-MS/MS) using a Waters UPLC (Waters UK, Herts, UK) coupled to an AB Sciex QTrap mass spectrometer (AB Sciex UK Ltd, Cheshire, UK) (5). Total folate was assessed as the sum of six folate forms methyl tetrahydrofolate, tetrahydrofolate, formyltetrahydrofolate, folic acid, 5,10 methenyltetrahydrofolate and an oxidation product of 5-methyltetrahydrofolate (MeFox). If any of the folate forms produced a result between the LoD and LoQ an imputed value equal to LoQ/√2 was used in the total calculation. If the concentration of any of the folate forms was less than the LoD or no peak was seen on the chromatogram then no value for this folate form was used in the total calculation. Stable isotope labelled internal standards were added during the extraction step; ^13^C_5_ 5-methyltetrahydrofolate, ^13^C_5_ folic acid, ^13^C_5_ tetrahydrofolate, ^13^C_5_ 5-formyltetrahydrofolate, ^13^C_5_ 5,10-methenyltetrahydrofolate and ^13^C_5_ MeFox (Merck & Cie, Schaffhausen, Switzerland).

The assay used solid phase extraction with phenyl columns. Separation was achieved with reversed phase ultra-performance liquid chromatography (UPLC) on a Waters ACQUITY UPLC® HSS T3 C8 1.7 µm 2.1 x 100 mm column (Waters UK, Herts, UK) at 300°C with a 49.5:40:10:0.5 water:methanol:acetonitrile:acetic acid isocratic mobile phase prior to mass spectrometry analysis. The assay was calibrated using in-house standards which were extracted alongside QC material and samples and accuracy checks performed using standard reference materials (NIST, Gaithersburg, Maryland, US).

- 1. **Whole blood folate and calculation of red cell folate**

At the time of this comparative study, samples from the UK National Diet and Nutrition Survey for whole blood folate analysis were mailed from the field to the MRC Elsie Widdowson Laboratory, Cambridge. Therefore, for this comparative study, cooled mailing of the samples described within the manuscript (“24-hour processed”) was compared to mailing of whole blood samples at ambient temperature. For each set of samples, prior to centrifugation, 100 µL of whole blood was added to 1 mL of 1% w/v ascorbic acid solution. Samples were subsequently frozen at -70°C before shipment on dry ice to the Centers for Disease Control and Prevention (CDC) (Atlanta, USA) for whole blood folate analysis.

Whole blood haemolysate specimens were analysed for total whole blood (WB) folate using the *Lactobacillus rhamnosus* microbiologic growth assay Pfeiffer et al. (6). Diluted specimen was added to an assay medium containing the microorganism and all of the nutrients necessary for the growth of the microorganism except for folate. The growth of *L. rhamnosus* is proportional to the amount of total folate present in the specimen and the total folate level was assessed by measuring the turbidity of the inoculated medium at 590 nm in a microplate reader.

Red blood cell (RBC) folate was calculated from WB folate concentration, serum folate concentration and, for this study, a surrogate haematocrit of 0.4 L/L, using the equation:

RBC folate = (WB folate - (serum folate x (1-haematocrit))) / haematocrit

Accuracy was established against the International Standard for Whole Blood Folate 95/528 (NIBSC, Hertfordshire, UK), and by successful participation in UKNEQAS Haematinics programme (http://www.ukneqas-haematinics.org.uk).

- 1. **Vitamin B-12**

Vitamin B-12 was measured at Addenbrooke’s Hospital, Cambridge on a Siemens ADVIA Centaur (Siemens Healthcare Limited, Camberley, Surrey, UK). The assay is a competitive immunoassay using direct chemiluminescent technology in which vitamin B-12 from the sample competes with vitamin B-12 labelled with acridinium ester, for a limited amount of purified intrinsic factor, which is covalently coupled to paramagnetic particles in the solid phase. The assay uses releasing agent (sodium hydroxide) and dithiothreitol (DTT) to release the vitamin B-12 from the endogenous binding proteins in the sample and cobinamide to prevent rebinding after the solid phase is added to the sample.

- 1. **Holo-transcobalamin (‘active’ vitamin B-12)**

Vitamin B-12 (cobalamin) is transported in the circulation bound to transcobalamin (TC) (10-30%) and to haptocorrin (HC) (70-90%). When TC and HC bind vitamin B-12 the resulting complexes are known as holotranscobalamin (holo-TC) and holohaptocorrin (holo-HC). Holo-TC is the only form of vitamin B-12 that can be taken up by cells in the body and may be a better marker of metabolic function than serum vitamin B-12 (7). The holo-TC assay is an enzyme-linked immunosorbent assay (ELISA) (Axis-Shield Diagnostics Ltd, Dundee, Scotland).

- 1. **Vitamin C**

The vitamin C assay was based on the procedure described by Vuilleumier & Keck (8). In the assay, vitamin C in the sample is converted to dehydroascorbic acid by ascorbate oxidase, followed by coupling of the resulting dehydroascorbate with o-phenylene diamine to form a fluorescent derivative quinoxaline. The formation of quinoxaline is linearly related to the amount of vitamin C in the sample. The assay was performed on the BMG Labtech FLUOstar Optima (BMG Labtech Ltd, Bucks, UK). The assay was calibrated using pure ascorbic acid (AnalaR grade, VWR International Limited, Leicestershire, UK) and external quality assessment provided by participation in Royal College of Pathologists of Australasia QA Program (RCPAQAP, Sydney, Australia).

**References for Supplemental Methods**

(1) Sowell AL, Huff DL, Yeager PR, Caudill SP, Gunter EW. Retinol, alpha-tocopherol, lutein/zeaxanthin, beta-cryptoxanthin, lycopene, alpha-carotene, trans-beta-carotene, and four retinyl esters in serum determined simultaneously by reversed-phase HPLC with multiwavelength detection. Clin Chem. 1994;40:411-6.

(2) Jones KS, Parkington DA, Cox LJ, Koulman A. Erythrocyte transketolase activity coefficient (ETKAC) assay protocol for the assessment of thiamine (vitamin B1) status. Ann N Y Acad Sci. 2020; Dec 22 (Epub ahead of print; DOI: 10.1111/nyas.14547.

(3) Glatzle D, Körner WF, Christeller S, Wiss O. Method for the detection of a biochemical riboflavin deficiency. Stimulation of NADPH2-dependent glutathione reductase from human erythrocytes by FAD in vitro. Investigations on the vitamin B2 status in healthly people and geriatric patients. Int Z Vitaminforsch. 1970;40:166-83.

(4) Rybak ME, Pfeiffer CM. Clinical analysis of vitamin B(6): determination of pyridoxal 5'-phosphate and 4-pyridoxic acid in human serum by reversed-phase high-performance liquid chromatography with chlorite postcolumn derivatization. Anal Biochem. 2004;333:336-44.

(5) Meadows S. Multiplex measurement of serum folate vitamers by UPLC-MS/MS. Methods Mol Biol. 2017;1546:245-256.

(6) Pfeiffer CM, Zhang M, Lacher DA, Molloy AM, Tamura T, Yetley EA, Picciano MF, Johnson CL. Comparison of serum and red blood cell folate microbiologic assays for national population surveys. J Nutr. 2011;141:1402-9.

(7) Allen LH, Miller JW, de Groot L, Rosenberg IH, Smith AD, Refsum H, Raiten DJ. Biomarkers of Nutrition for Development (BOND): Vitamin B-12 Review. J Nutr. 2018;148(suppl_4):1995S-2027S.

(8) Vuilleumier JP, Keck E. Fluorometric assay of vitamin C in biological materials using a centrifugal analyser with fluorescence attachment. J Micronutr Anal. 1989; 5: 25-34.
