# Supplementary material for: Delayed Processing of Chilled Whole Blood for 24 Hours Does Not Affect the Concentration of the Majority of Micronutrient Status Biomarkers
Source: J Nutr. 2021 Sep 6;151(11):3524–32. doi: 10.1093/jn/nxab267 (PMC8564691; doi:10.1093/jn/nxab267)
Supplement: nxab267_Supplemental_Files [file nxab267_Supplemental_Files.zip › JN-2021-0500_R1 Supplemental data results.docx]

**Supplemental Results**

Supplemental Table S1. Sample sizes for each analyte, treatment and sample type……..…2

Supplemental results for plasma/serum folate vitamers…..……………………………………..3

Supplemental Table S2. Geometric mean percent difference of total folate and folate………3 vitamers concentration between whole blood samples processed within 2 hours or 24
hours

Supplemental Table S3. Geometric mean concentration of total folate and folate vitamers…4 (95% CI) by treatment (processed within 2 hours or 24 hours) and sample type (EDTA, LH
or serum)

Supplemental Figures S1 – S25. Deming regression plots for each analyte…...………..5 – 18

Supplemental results for red blood cell folate ………………………….………………………..19

Supplemental Figure S26. Deming regression plots for red blood cell folate………………...19

**Table S1. Sample sizes for each analyte, treatment and sample type1**

|  |  |  |  |  |  |  |
| --- | --- | --- | --- | --- | --- | --- |
|  | 2-hour processed | | | 24-hour processed | | |
|  | EDTA | LH | Serum | EDTA | LH | Serum |
|  | n | | | | | |
| **Clinical markers** |  |  |  |  |  |  |
| C-reactive protein | 14 | 14 | 16 | 14 | 15 | 16 |
| Ferritin | 14 | 14 | 16 | 14 | 15 | 16 |
| Creatinine | 14 | 14 | 16 | 14 | 15 | 16 |
| Triglycerides | 14 | 14 | 16 | 14 | 15 | 16 |
| HDL cholesterol | 14 | 14 | 16 | 14 | 15 | 16 |
| Total cholesterol | 14 | 14 | 16 | 14 | 15 | 16 |
| **Fat-soluble vitamins** |  |  |  |  |  |  |
| 25-hydroxyvitamin D | 14 | 15 | 16 | 14 | 15 | 16 |
| Retinol | 16 | 15 | 16 | 14 | 15 | 16 |
| α-tocopherol | 14 | 15 | 16 | 14 | 15 | 16 |
| γ-tocopherol | 14 | 15 | 16 | 14 | 15 | 16 |
| Lutein | 14 | 15 | 16 | 14 | 15 | 16 |
| β-cryptoxanthin | 13 | 14 | 13 | 13 | 14 | 14 |
| Lycopene | 14 | 15 | 16 | 14 | 15 | 16 |
| α-carotene | 10 | 10 | 10 | 10 | 10 | 11 |
| β -carotene | 14 | 15 | 16 | 14 | 15 | 16 |
| **Minerals** |  |  |  |  |  |  |
| Selenium | 14 | 14 | 14 | 14 | 15 | 15 |
| Zinc | 14 | 14 | 14 | 14 | 15 | 15 |
| **Water-soluble vitamins** |  |  |  |  |  |  |
| ETKAC (thiamin) | 15 | 16 | - | 15 | 16 | - |
| EGRAC (riboflavin) | 15 | 14 | - | 15 | 16 | - |
| PLP (vitamin B-6) | 14 | 15 | 16 | 14 | 15 | 16 |
| PA (vitamin B-6) | 14 | 15 | 16 | 14 | 15 | 16 |
| Total folate | 14 | 15 | 16 | 14 | 15 | 16 |
| Red blood cell folate2 | 15 | 14 | - | 15 | 15 | - |
| Vitamin B-12 | 14 | 15 | 16 | 14 | 15 | 16 |
| Holotranscobalamin | 10 | 11 | 12 | 10 | 11 | 12 |
| Vitamin C | 14 | 16 | 16 | 14 | 16 | 16 |

1 24-hour processed samples were mailed, cooled, overnight after collection and processed on arrival at the laboratory. All analytes were measured in plasma or serum except for ETKAC and EGRAC (measured in saline washed red blood cells) and red blood cell folate (measured in whole blood).

2 Red blood cell folate concentrations were compared between whole blood samples mailed either at ambient temperature or cooled (as described in the manuscript for the 24-hour processed samples). See page 19 of Supplemental Results for further details.

Abbreviations: EDTA, ethylenediaminetetraacetic acid plasma; EGRAC, erythrocyte glutathione reductase activity coefficient; ETKAC, erythrocyte transketolase activity coefficient; LH, lithium heparin plasma; PA, 4-pyridoxic acid; PLP, pyridoxal-5-phosphate.

**Results for plasma/serum folate vitamers**

Table S2 shows the geometric mean percent difference between 2- and 24-hour processed samples for the folate vitamers that contribute to the calculation of plasma/serum total folate. In EDTA plasma, 5-methyltetrahydrofolate (5-MTHF) concentration was lower and the MeFox (pyrazino-s-triazine derivative of 4α-hydroxy-5-methyl tetrahydrofolate) concentration higher in 24-hour processed samples. 5-MTHF was also lower in 24-processed LH plasma samples, although the difference was smaller than for EDTA plasma.

Table S3 contains the geometric mean concentrations for the folate vitamers by treatment and tube type. Folate vitamer concentrations were variable amongst the tube types, particularly in the 24-hour processed samples, although folic acid and tetrahydrofolate (THF) concentrations were low (< 1nmol/L) across all tubes and the data are less reliable. For THF in particular, concentrations in 20 of the 90 samples measured were less than the limit of quantitation (0.18 nmol/L). For both sample treatments, and consistent with the pattern for total folate, 5-MTHF was lower in plasma from EDTA tubes than other sample types. In 24-hour processed samples, 5-MTHF was also lower in plasma from LH tubes than in serum. In 2-hour processed samples, and following the pattern for total folate, 5-MTHF was lower in whole blood collected in EDTA tubes compared with LH or serum tubes. There were differences in MeFox concentration between tube types, with the highest concentration in the 24-hour processed EDTA samples. Folic acid concentration was significantly lower in plasma compared to the serum for 24-hour processed samples although the numerical difference was small.

**Table S2. Geometric mean percent difference of total folate and folate vitamer concentration between whole blood samples processed within 2 hours or after 24 hours1,2,3**

|  | **Geometric mean percent difference (95% CI) from 2-hour processing** | | |
| --- | --- | --- | --- |
| **Analyte** | **Sample type** | | |
|  | EDTA | LH | Serum |
| Total folate4 | -2 (-5, 1) | -2 (-5, 1) | 1 (-2, 4) |
| 5-MTHF | -9 (-13, -5)* | -4 (-8, -0.4)* | -2 (-6, 3) |
| MeFox | 44 (31, 60)* | 3 (-6, 13) | -4 (-5, 15) |
| Folic acid | 0 (-18, 22) | -3 (-20, 17) | 5 (-13, 25) |
| THF | 38 (-27, 161) | 8 (-41, 100) | 95 (7, 254)* |
| 5,10-MTHF5 | - | - | - |
| 5-FTHF5 | - | - | - |

1 24-hour processed samples were mailed, cooled, overnight after collection and processed on arrival at the laboratory. Analytes were measured in plasma or serum by LC-MS/MS 2 * indicates a significant concentration difference (P<0.05) from samples that underwent processing within 2 hours of collection
3 Percent difference was calculated from linear mixed model with random effect of participant ID and fixed effects of treatment and sample type.
4 Total folate was assessed as the sum of six folate forms: 5-MTHF, THF, 5-FTHF, folic acid, 5,10-MTHF and oxidation product of 5-MTHF (MeFox)
5 All concentrations were below the LOQ

Abbreviations: 5-FTHF, 5-formyltetrahydrofolate; 5-MTHF, 5-methyl tetrahydrofolate; 5,10-MTHF, 5,10-methenyltetrahydrofolate; EDTA, ethylenediaminetetraacetic acid plasma; LC-MS/MS; liquid chromatography tandem mass spectrometry; LH, lithium heparin plasma; MeFox, pyrazino-s-triazine derivative of 4α-hydroxy-5-methyl tetrahydrofolate, an oxidation product of 5-methyl tetrahydrofolate; THF, tetrahydrofolate

**Table S3. Geometric mean concentration of total folate and folate vitamers (95% CI) by treatment (processed within 2 hours or 24 hours) and sample type (EDTA, LH or serum) 1,2**

| **Analyte** | **2-hour processing** | | |  | **24-hour processing** | | |
| --- | --- | --- | --- | --- | --- | --- | --- |
|  | **Sample type** | | |  | **Sample type** | | |
|  | EDTA | LH | Serum |  | EDTA | LH | Serum |
| Total folate, nmol/L3 | 16.2 (12.3, 21.4) a | 17.0 (12.9, 22.5) b | 17.0 (12.9, 22.4) b |  | 15.9 (12.1, 20.9) a | 16.7 (12.6, 21.9) b | 17.2 (13.0, 22.6) c |
| 5-MTHF, nmol/L | 13.2  (9.7, 17.8) a | 14.2  (10.5, 19.2) b | 14.4  (10.6, 19.4) b |  | 11.9 (8.8, 16.1) *,a | 13.5  (10.0, 18.3)*,b | 14.1  (10.4, 19.1) c |
| MeFox, nmol/L | 1.6  (1.2, 2.1) a,b | 1.5  (1.2, 2.0) a | 1.7  (1.3, 2.2) b |  | 2.3  (1.7, 3.1)*,a | 1.6  (1.2, 2.1) b | 1.8  (1.3, 2.3) c |
| Folic acid, nmol/L | 0.15 (0.08, 0.27) | 0.16 (0.09, 0.28) | 0.18 (0.10, 0.32) |  | 0.15 (0.08, 0.27) a | 0.15 (0.08, 0.27) a | 0.18 (0.10, 0.33) b |
| THF, nmol/L | 0.64 (0.39, 1.06) a | 0.46 (0.28, 0.74) a | 0.17 (0.11, 0.28) b |  | 0.89  (0.54, 1.46) a | 0.50 (0.30, 0.81) a,b | 0.33 (0.21, 0.54)*,b |
| 5,10-MTHF, nmol/L4 | - | - | - |  | - | - | - |
| 5-FTHF, nmol/L4 | - | - | - |  | - | - | - |

1 24-hour processed samples were mailed, cooled, overnight after collection and processed on arrival at the laboratory. Analytes were measured in plasma or serum by LC-MS/MS 2* indicates significantly different from 2-hour processed sample. Geometric means with uncommon letters are significantly different (P<0.05) from other sample type within treatment (2-hour or 24-hour processing). 24-hour processed samples were mailed, cooled, overnight after collection and processed on arrival at the laboratory.
3 Total folate was assessed as the sum of six folate forms: 5-MTHF, THF, 5-FTHF, folic acid, 5,10-MTHF and oxidation product of 5-MTHF (MeFox)
4 All concentrations were below the LOQ

Abbreviations: 5-FTHF, 5-formyltetrahydrofolate; 5-MTHF, 5-methyl tetrahydrofolate; 5,10-MTHF, 5,10-methenyltetrahydrofolate; EDTA, ethylenediaminetetraacetic acid plasma; LC-MS/MS; liquid chromatography tandem mass spectrometry; LH, lithium heparin plasma; MeFox, pyrazino-s-triazine derivative of 4α-hydroxy-5-methyl tetrahydrofolate, an oxidation product of 5-methyl tetrahydrofolate; THF, tetrahydrofolate

**Figures S1 – S25: Deming regression plots**

The charts contained with Figures S1 – S25, show Deming regression analysis for 2-hour vs. 24-hour processing of whole blood samples and for comparison of sample type.

In summary, six tubes of blood (two each of EDTA, LH and serum) were collected from 16 participants. Samples were either processed within 2 hours of collection (“2-hours processed”) or mailed, chilled, using a next-day (before 9 am) delivery service (www.royalmail.com) using an ‘Icertech’ insulated foil-box and ‘Easi-chill’ cool pack (Icertech, Wrexham, UK), (“24-hour processed”). Samples were processed according to standardised protocols as described in the manuscript and detailed in the Supplementary Methods. The methods were those used in the UK National Diet and Nutrition Survey Rolling Programme (https://www.gov.uk/government/statistics/ndns-results-from-years-9-to-11-2016-to-2017-and-2018-to-2019).

The charts below show the results of Deming regression performed in Graphpad Version 9 (Graphpad Software, San Diego, CA) including the equation for each line to 3 significant figures. Plots show (a) 24-hour processed samples vs. samples processed within 2 hours (top left panel) and (b) and (c) alternative sample type against the laboratory routine sample sample type 2 hours (bottom left panel) and 24 hours (bottom right panel). The dashed line is the line of equality (x=y).

**Figure S1.** Deming regression plots for C-reactive protein (CRP)**.** (A)24-hour processed samples *vs.* 2-hour processed samples. Alternative sample type against the sample type routinely used at the time of the study for (B) 2-hour processed samples and (C) 24-hour processed samples. The dashed line is the line of equality (x=y). Abbreviations: EDTA, ethylenediaminetetraacetic acid; LH, lithium heparin.**Figure S2.** Deming regression plots for ferritin**.** (A)24-hour processed samples *vs.* 2-hour processed samples. Alternative sample type against the sample type routinely used for analysis at the time of the study for (B) 2-hour processed samples and (C) 24-hour processed samples. The dashed line is the line of equality (x=y). Abbreviations: EDTA, ethylenediaminetetraacetic acid; LH, lithium heparin.

A

B

C

A

B

C

**Figure S3.** Deming regression plots for creatinine**.** (A)24-hour processed samples *vs.* 2-hour processed samples. Alternative sample type against the sample type routinely used for analysis at the time of the study for (B) 2-hour processed samples and (C) 24-hour processed samples. The dashed line is the line of equality (x=y). Abbreviations: EDTA, ethylenediaminetetraacetic acid; LH, lithium heparin.
**Figure S4.** Deming regression plots for triglycerides**.** (A)24-hour processed samples *vs.* 2-hour processed samples. Alternative sample type against the sample type routinely used for analysis at the time of the study for (B) 2-hour processed samples and (C) 24-hour processed samples. The dashed line is the line of equality (x=y). Abbreviations: EDTA, ethylenediaminetetraacetic acid; LH, lithium heparin.

A

B

C

A

B

C

**Figure S5.** Deming regression plots for total cholesterol**.** (A)24-hour processed samples *vs.* 2-hour processed samples. Alternative sample type against the sample type routinely used for analysis at the time of the study for (B) 2-hour processed samples and (C) 24-hour processed samples. The dashed line is the line of equality (x=y). Abbreviations: EDTA, ethylenediaminetetraacetic acid; LH, lithium heparin. **Figure S6.** Deming regression plots for HDL cholesterol**.** (A)24-hour processed samples *vs.* 2-hour processed samples. Alternative sample type against the sample type routinely used for analysis at the time of the study for (B) 2-hour processed samples and (C) 24-hour processed samples. The dashed line is the line of equality (x=y). Abbreviations: EDTA, ethylenediaminetetraacetic acid; LH, lithium heparin.

A

B

C

A

B

C

**Figure S7.** Deming regression plots for 25-hydroxyvitamin D**.** (A)24-hour processed samples *vs.* 2-hour processed samples. Alternative sample type against the sample type routinely used for analysis at the time of the study for (B) 2-hour processed samples and (C) 24-hour processed samples. The dashed line is the line of equality (x=y). Abbreviations: EDTA, ethylenediaminetetraacetic acid; LH, lithium heparin. **Figure S8.** Deming regression plots for retinol**.** (A)24-hour processed samples *vs.* 2-hour processed samples. Alternative sample type against the sample type routinely used for analysis at the time of the study for (B) 2-hour processed samples and (C) 24-hour processed samples. The dashed line is the line of equality (x=y). Abbreviations: EDTA, ethylenediaminetetraacetic acid; LH, lithium heparin.

A

B

C

A

B

C

**Figure S9.** Deming regression plots for α-tocopherol.(A)24-hour processed samples *vs.* 2-hour processed samples. Alternative sample type against the sample type routinely used for analysis at the time of the study for (B) 2-hour processed samples and (C) 24-hour processed samples. The dashed line is the line of equality (x=y). Abbreviations: EDTA, ethylenediaminetetraacetic acid; LH, lithium heparin. **Figure S10.** Deming regression plots for γ-tocopherol.(A)24-hour processed samples *vs.* 2-hour processed samples. Alternative sample type against the sample type routinely used for analysis at the time of the study for (B) 2-hour processed samples and (C) 24-hour processed samples. The dashed line is the line of equality (x=y). Abbreviations: EDTA, ethylenediaminetetraacetic acid; LH, lithium heparin.

A

B

C

A

B

C

**Figure S11.** Deming regression plots for lutein.(A)24-hour processed samples *vs.* 2-hour processed samples. Alternative sample type against the sample type routinely used for analysis at the time of the study for (B) 2-hour processed samples and (C) 24-hour processed samples. The dashed line is the line of equality (x=y). Abbreviations: EDTA, ethylenediaminetetraacetic acid; LH, lithium heparin.
**Figure S12.** Deming regression plots for β-cryptoxanthin.(A)24-hour processed samples *vs.* 2-hour processed samples. Alternative sample type against the sample type routinely used for analysis at the time of the study for (B) 2-hour processed samples and (C) 24-hour processed samples. The dashed line is the line of equality (x=y). Abbreviations: EDTA, ethylenediaminetetraacetic acid; LH, lithium heparin.

A

B

C

A

B

C

**Figure S13.** Deming regression plots for lycopene.(A)24-hour processed samples *vs.* 2-hour processed samples. Alternative sample type against the sample type routinely used for analysis at the time of the study for (B) 2-hour processed samples and (C) 24-hour processed samples. The dashed line is the line of equality (x=y). Abbreviations: EDTA, ethylenediaminetetraacetic acid; LH, lithium heparin.

**Figure S14.** Deming regression plots for α-carotene.(A)24-hour processed samples *vs.* 2-hour processed samples. Alternative sample type against the sample type routinely used for analysis at the time of the study for (B) 2-hour processed samples and (C) 24-hour processed samples. The dashed line is the line of equality (x=y). Abbreviations: EDTA, ethylenediaminetetraacetic acid; LH, lithium heparin. **Figure S15.** Deming regression plots for β-carotene.(A)24-hour processed samples *vs.* 2-hour processed samples. Alternative sample type against the sample type routinely used for analysis at the time of the study for (B) 2-hour processed samples and (C) 24-hour processed samples. The dashed line is the line of equality (x=y). Abbreviations: EDTA, ethylenediaminetetraacetic acid; LH, lithium heparin.
 **Figure S16.** Deming regression plots for selenium.(A)24-hour processed samples *vs.* 2-hour processed samples. Alternative sample type against the sample type routinely used for analysis at the time of the study for (B) 2-hour processed samples and (C) 24-hour processed samples. The dashed line is the line of equality (x=y). Abbreviations: EDTA, ethylenediaminetetraacetic acid; LH, lithium heparin.

A

B

C

A

B

C

A

B

C

A

B

C

A

B

C

**Figure S17.** Deming regression plots for zinc.(A)24-hour processed samples *vs.* 2-hour processed samples. Alternative sample type against the sample type routinely used for analysis at the time of the study for (B) 2-hour processed samples and (C) 24-hour processed samples. The dashed line is the line of equality (x=y). Abbreviations: EDTA, ethylenediaminetetraacetic acid; LH, lithium heparin.

A

B

C

**Figure S18.** Deming regression plots for erythrocyte transketolase activity coefficient (ETKAC).(A)24-hour processed samples *vs.* 2-hour processed samples. Alternative sample type against the sample type routinely used for analysis at the time of the study for (B) 2-hour processed samples and (C) 24-hour processed samples. The dashed line is the line of equality (x=y). Abbreviations: ETDA, ethylenediaminetetraacetic acid; LH, lithium heparin.

A

B

C

**Figure S19.** Deming regression plots for erythrocyte glutathione reductase activity coefficient (EGRAC).(A)24-hour processed samples *vs.* 2-hour processed samples. Alternative sample type against the sample type routinely used for analysis at the time of the study for (B) 2-hour processed samples and (C) 24-hour processed samples. The dashed line is the line of equality (x=y). Abbreviations: ETDA, ethylenediaminetetraacetic acid; LH, lithium heparin.

A

B

C

**Figure S20.** Deming regression plots for pyridoxal-5-phosphate (PLP) [vitamin B-6].(A)24-hour processed samples *vs.* 2-hour processed samples. Alternative sample type against the sample type routinely used for analysis at the time of the study for (B) 2-hour processed samples and (C) 24-hour processed samples. The dashed line is the line of equality (x=y). Abbreviations: ETDA, ethylenediaminetetraacetic acid; LH, lithium heparin.

A

B

C

**Figure S21.** Deming regression plots for pyridoxic acid (PA) [vitamin B-6].(A)24-hour processed samples *vs.* 2-hour processed samples. Alternative sample type against the sample type routinely used for analysis at the time of the study for (B) 2-hour processed samples and (C) 24-hour processed samples. The dashed line is the line of equality (x=y). Abbreviations: ETDA, ethylenediaminetetraacetic acid; LH, lithium heparin.

A

B

C

B

C

A

**Figure S22.** Deming regression plots for total folate (A)24-hour processed samples *vs.* 2-hour processed samples. Alternative sample type against the sample type routinely used for analysis at the time of the study for (B) 2-hour processed samples and (C) 24-hour processed samples. The dashed line is the line of equality (x=y). Abbreviations: ETDA, ethylenediaminetetraacetic acid; LH, lithium heparin.

**Figure S23.** Deming regression plots for vitamin B-12 (A)24-hour processed samples *vs.* 2-hour processed samples. Alternative sample type against the sample type routinely used for analysis at the time of the study for (B) 2-hour processed samples and (C) 24-hour processed samples. The dashed line is the line of equality (x=y). Abbreviations: ETDA, ethylenediaminetetraacetic acid; LH, lithium heparin.

A

B

C

**Figure S24.** Deming regression plots for holo-transcobalamin [vitamin B-12] (A)24-hour processed samples *vs.* 2-hour processed samples. Alternative sample type against the sample type routinely used for analysis at the time of the study for (B) 2-hour processed samples and (C) 24-hour processed samples. The dashed line is the line of equality (x=y). Abbreviations: ETDA, ethylenediaminetetraacetic acid; LH, lithium heparin.

A

B

C

**Figure S25.** Deming regression plots for vitamin C (A)24-hour processed samples *vs.* 2-hour processed samples. Alternative sample type against the sample type routinely used for analysis at the time of the study for (B) 2-hour processed samples and (C) 24-hour processed samples. The dashed line is the line of equality (x=y). Abbreviations: ETDA, ethylenediaminetetraacetic acid; LH, lithium heparin.

A

B

C

**Results for red blood cell folate**

Red blood cell folate concentrations were compared between whole blood samples mailed either at ambient temperature or cooled (as described in the manuscript for the 24-hour processed samples). Further detail and the analytical methods are contained within the Supplemental Methods.

*Effect of ambient or cooled mailing on red blood cell folate*

There was no significant difference in red blood cell folate concentration between ambient and cooled mailing of whole blood samples. Geometric mean (95% CI) percent difference from ambient posting was for the EDTA sample -1 (-7, 6)% and for the LH sample -4 (-10,3)%. Red cell folate was not measured in whole blood collected in serum tubes.

*Effect of sample type*

We observed no significant difference in red blood cell folate concentration between sample type for either the ambient mailed or cooled mailed samples. For ambient mailed samples, the geometric means (95% CI) were 280 (229, 341) and 285 (233, 348) nmol/L for EDTA and LH, respectively. For cooled, mailed samples concentrations were 277 (227, 338) and 273 (224, 333) nmol/L for EDTA and LH, respectively.

A

B

C

**Figure S26.** Deming regression plots for red blood cell folate (A)Cooled, mailed samples *vs.*ambient mailed samples. Alternative sample type against the sample type routinely used for analysis at the time of the study for (B) ambient mailed samples and (C) cooled, mailed samples. The dashed line is the line of equality (x=y). Abbreviations: ETDA, ethylenediaminetetraacetic acid; LH, lithium heparin.
